# A comprehensive study on seroprevalence of bluetongue virus in Haryana state of India

**DOI:** 10.14202/vetworld.2017.1464-1470

**Published:** 2017-12-13

**Authors:** Sushila Maan, Anuj Tiwari, Deepika Chaudhary, Anita Dalal, Nitish Bansal, Vinay Kumar, Kanisht Batra, Aman Kumar, Naresh Kumar Kakker, Narender Singh Maan

**Affiliations:** 1 Department of Animal Biotechnology, College of Veterinary Sciences, Lala Lajpat Rai University of Veterinary and Animal Sciences, Hisar - 125 004, Haryana, India; 2 Department of Veterinary Microbiology, G. B. Pant University of Agriculture and Technology, Pantnagar - 263 145, Uttarakhand, India; 3 Department of Veterinary Microbiology, College of Veterinary Sciences, Lala Lajpat Rai University of Veterinary and Animal Sciences, Hisar - 125 004, Haryana, India; 4 Department of Animal Nutrition, College of Veterinary Sciences, Lala Lajpat Rai University of Veterinary and Animal Sciences, Hisar - 125 004, Haryana, India

**Keywords:** bluetongue, bluetongue virus, buffalo, cattle, competitive enzyme-linked immunosorbent assay, Haryana, India, serology

## Abstract

**Aim:**

The aim of present study was to determine seroprevalence of bluetongue virus (BTV) in Haryana state of India.

**Materials and Methods:**

A total of 803 serum samples, 408 of cattle and 395 of buffalo origin, respectively, were collected from different villages of Haryana. Sampling was done randomly to obtain unbiased results. The samples were evaluated by a competitive enzyme-linked immunosorbent assay for the presence of BTV antibodies.

**Results:**

Overall seroprevalence of BTV antibody in cattle and buffaloes for all 21 districts of Haryana state was found to be 75.49% and 92.91%, respectively. The prevalence of BTV in different agroclimatic zones ranged between 72-77% and 90-94% for cattle and buffalo, respectively. In buffaloes, the BTV seroprevalence was comparatively higher than in cattle.

**Conclusion:**

The study showed that BTV is circulating in cattle and buffalo populations in the Northern part of India.

## Introduction

Bluetongue (BT) is an arthropod-borne disease of wild and domestic animals caused by bluetongue virus (BTV) of genus *Orbivirus* in the *Reoviridae* family [1,2]. The disease is characterized by fever, facial edema, hemorrhages, and ulceration on the oral mucosa and coronitis. Cattle and buffaloes are sub-clinically affected and are carriers of BTV. Even though this true for many strains of the virus, however, during the European outbreak of BT caused by BTV-8 in 2006, the cattle were severely affected [3]. The clinical signs in BTV-8 affected sheep flocks and cattle herds in 2007 and 2006 were similar.

BTV is not contagious, and it is transmitted by *Culicoides* vector midges [4,5]. In India, the disease is endemic particularly in Southern states namely, Tamil Nadu, Karnataka, Kerala, and Andhra Pradesh. To date, 27 serotypes of this virus have been reported worldwide; however, there are further putative serotypes reported recently [6-10]. There is a low level of cross-protection among different serotypes, thus creating difficulties when designing of vaccines, vaccination strategies, and planning control measures [11]. Fifteen BTV serotypes have been isolated in India (BTV-1, -2, -3, -4, -5, -8, -9, -10, -12, -16, -17, -18, -21, -23, and -24), eleven of these in the past decade, with serological evidence of presence of eight more (BTV-6, -7, -11, -13, -14, -15, -19, and -20) [12]. BT outbreaks result in an estimated 3 billion USD/annum losses across the globe, both directly and indirectly [13,14]. Although there are no precise figures for economic losses due to BT in India, a study published in 2009 assessed the economic losses due to important diseases of sheep in India between 1991 and 2005, and BT was found to cause more economic devastation than foot and mouth disease (FMD), peste des petits ruminants, sheep and goat pox, anthrax, fascioliasis/distomatosis, and enterotoxemia [15]. The greatest direct losses to farmers due to BT occurred in 2005, amounting to approximately 231 million rupees [16]. Direct losses are a result of mortality and decline in production of affected animals. Indirect losses are due to trade embargoes and animal movement restrictions [1].

Due to its huge economic importance, it is paramount to detect BTV infection both in endemic and BTV-free countries. Complement fixation test, agar gel immunodiffusion (AGID), and competitive enzyme-linked immunosorbent assay (c-ELISA) are the OIE recommended methods for BT testing for international trade [17]. Of these 3 tests, the c-ELISA has proven to be highly sensitive. It can be used to detect antibodies raised against all BTV serotypes [15]. The specificity of this test is due to the use of monoclonal anti-VP7 which can distinguish the BT serogroup from other orbivirus serogroups, for example, epizootic hemorrhagic disease virus (EHDV) [18-20]. It has been found that the diagnostic sensitivity and specificity of c-ELISA is 87.8% (85.10-91.10) and 98.2% (96.30-99.60), respectively [21]. Therefore, the antibody detecting VP7-based c-ELISA was employed in this study to determine the seroprevalence of BTV in Haryana state of India.

In India, a few studies have been conducted on seroprevalence of BT, but their results do not have much significance due to testing of less/small number of samples. This is the first substantive seroprevalence study of BT in Haryana state based on random sampling and testing of a large number of serum samples from cattle and buffaloes.

## Materials and Methods

### Ethical approval

Ethical approval from Institutional Animal Ethics Committee was not required as no part of research had been carried out in live laboratory or domestic animals.

### Animals

The blood samples were collected from 803 animals (cattle and buffaloes) from 80 villages of 21 district of Haryana. The details of animals are given inTable-1.

**Table-1:** Details of animals from which samples were collected.

District	Animal ID	Species	Adult	Heifers	Young	Female	Male
A: Buffalo							
Rohtak	20	Buffalo	20			16	4
Faridabad	11	Buffalo	9	2		11	
Jind	20	Buffalo	20			13	7
Gurgaon	20	Buffalo	14	6		20	
Kurukshetra	12	Buffalo	12			11	1
Rewari	30	Buffalo	13	11	6	28	2
Sirsa	20	Buffalo	20			20	
Panipat	20	Buffalo	17	3		20	
Kaithal	10	Buffalo	8	2		8	2
Mewat	20	Buffalo	12	8		17	3
Jhajjar	20	Buffalo	14	6		19	1
Fatehabad	20	Buffalo	18	2		20	
Bhiwani	20	Buffalo	20			20	
Hisar	12	Buffalo	11	1		12	
Ambala	20	Buffalo	20			20	
Sonepat	20	Buffalo	20			20	
Palwal	20	Buffalo	17	2	1	19	1
Panchkula	20	Buffalo	18	2		19	1
Yamunanagar	20	Buffalo	13	5	2	17	3
Mahendragarh	20	Buffalo	15	5		20	
Karnal	20	Buffalo	18	2		18	2
Total	395	Buffalo	329	57	9	368	27
B: Cattle							
Faridabad	10	Cattle	7	2	1	10	
Sirsa	20	Cattle	17	3		20	
Rewari	10	Cattle	6	2	2	8	2
Panipat	20	Cattle	18	2		20	
Kaithal	30	Cattle	26	2	2	30	
Ambala	20	Cattle	19	1		18	2
Sonepat	20	Cattle	16	3	1	18	2
Palwal	20	Cattle	17	2	1	18	2
Panchkula	20	Cattle	17	3		19	1
Yamunanagar	12	Cattle	12			11	1
Mewat	20	Cattle	14	6		19	1
Jhajjar	20	Cattle	10	5	5	12	8
Fatehabad	20	Cattle	20			20	
Bhiwani	19	Cattle	19			19	
Hisar	16	Cattle	10	6		12	4
Karnal	20	Cattle	18	2		19	1
Mahendegarh	20	Cattle	9	10	1	20	
Rohtak	20	Cattle	20			12	8
Faridabad	19	Cattle	17	2		19	
Jind	20	Cattle	19	1		19	1
Gurgaon	20	Cattle	16	4		19	1
Kurukshetra	12	Cattle	10	2		12	
Total	408	Cattle	337	58	13	374	34

Young 0-1 year, Heifers 1-3 Year, Adult >3 Year

### Study area

Haryana is one of the northern states of India situated between 27° 37’ to 30° 35’ latitude and between 74° 28’ to 77° 36’ longitude and with an altitude between 700 and 3600 ft above sea level. Haryana is primarily an agricultural state. The general slope of the terrain is from north-east to south-west and west with an exception in the south in Bhiwani, Mahendragarh, Rewari, and Gurgaon districts where the slope is toward north. Most of the year, the climate of Haryana is very hot in summer (May to June) and markedly cold in winter (December and January) where as in between are the pleasant months of spring. The rainfall in the region is low and erratic except in parts of Karnal, Kurukshetra, and Ambala districts ranging from 300 mm in the southwest to 1100 mm in the northeast of the state. The samples were collected throughout the year.

### Serum samples

Serum was separated from 5 ml of blood collected from cattle and buffaloes, which was stored at −20°C until further use. A total of 803 serum samples, 408 from cattle and 395 from buffaloes, were collected through RRC-FMD from 80 villages of Haryana in 2014. These blood samples were taken from animals in the field, by qualified veterinarians, as part of normal veterinary care and diagnostic testing procedures in the Haryana, India. The statistical model is described as follows.

### Statistical model for random sampling and sample size determination

To conduct serosurveillance studies in Haryana state, a model was designed for random sampling using SPSS software. The number of samples to be tested was calculated as described below. Villages having cattle/buffalo population more than 200 were taken for statistical analysis. The prevalence of BT was considered as 50% with 95% confidence interval. Using these values, random sampling model was designed. The above values along with the animal population data as per 19^th^ livestock census of 2012 for all villages in Haryana were used to obtain the number and name of villages for random sampling (http://pashudhanharyana.gov.in/html/pdf%20&%20downloads/LC%202012/Districtwise%20Livestock%20Census%202012.pdf).

### c-ELISA

The c-ELISA kits were procured from the Veterinary Medical Research and Development, USA. The ELISA was performed as per the manufacturer’s instructions. Briefly, 25 µl of neat serum, positive control, and negative control were added to antigen (VP7) pre-coated plates in duplicates and test samples in a single well. These plates were then firmly tapped 14 times on the long sides and incubated for 15 min at room temperature (RT). 25 µl of antibody peroxidase conjugate was then added to each well, tapped firmly, and further incubated for 15 min at RT. Plates were then washed 3 times using wash solution, and remaining sera was removed by striking the inverted plates 4 times on a clean paper towel. Thereafter, 50 µl of substrate was added, and plates were tapped firmly and incubated for 10 min at RT. 50 µl of stop solution was added, and immediately, the plates were read in a microplate spectrophotometer at 620 nm. The plates were deemed pass if the mean of negative control optical density (OD) was 0.300-2.00 and mean of the positive control was <50% of the mean of negative control. Test samples were considered positive if they produce OD <50% of the mean of negative controls and negative if they produce OD >50% of the mean of negative controls.

## Results

**Figure-1:**
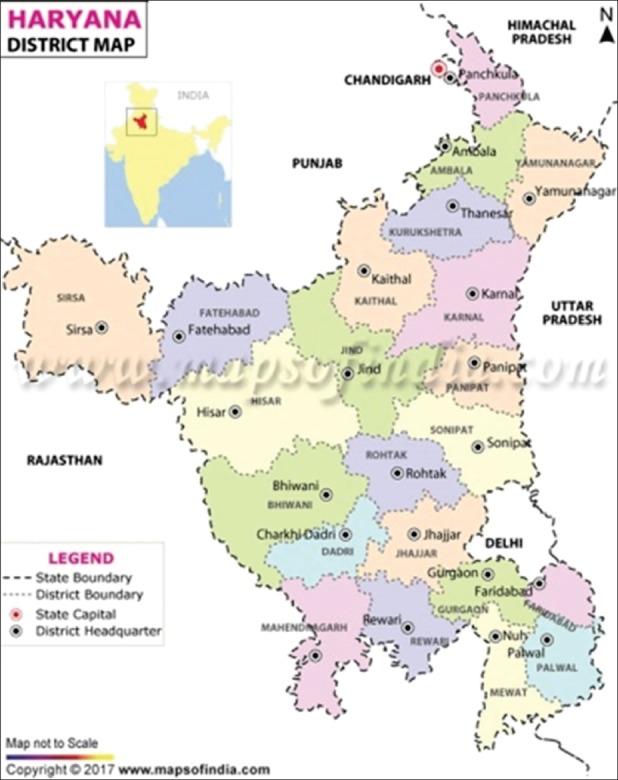
Study area - Haryana state districts depicted on India map.

**Figure-2:**
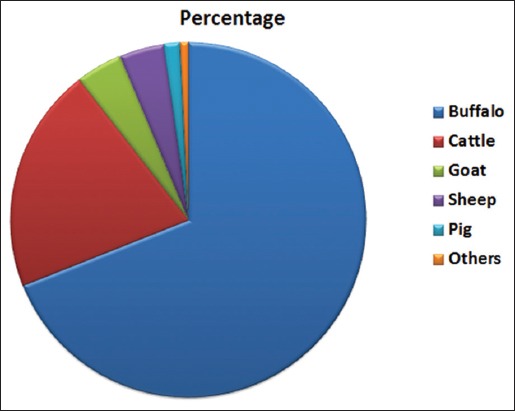
Distribution of livestock in Haryana. Adapted from 19^th^ livestock census 2012, chapter 14, Haryana.

Based on the random sampling mode, a total of 803 serum samples, 408 from cattle and 395 from buffaloes, were collected from 80 different villages of 21 districts of Haryana (Figure-1). In Haryana, the domestic animal population is dominated by buffaloes (6.08 million - 69%) and cattle (1.80 million - 20.50%), whereas sheep (0.36 million - 4.11%), goats (0.36 million - 4.19%), pigs only 1.44%, and remaining 0.76% constituted by other species as shown in pie chart (Figure-2).

Different districts of Haryana are divided into two agroclimatic zones, i.e. eastern and western zones as shown in Haryana map Figure-1 and Table-2. The prevalence of BTV in different agroclimatic zones ranged between 72-77% (Table-3) and 90-94% (Table-4) for cattle and buffalo, respectively. The prevalence of BTV antibodies in cattle and buffalo per district is shown in Table-5.

**Table-2:** List of districts in different agroclimatic zones of Haryana.

Eastern zone	Western zone
Ambala	Bhiwani
Faridabad	Fatehabad
Gurugram	Hisar
Jhajjar	Mahendergarh
Kaithal	Rewari
Karnal	Sirsa
Kurukshetra	Charkhi Dadri
Panipat	
Rohtak	
Sonipat	
Yamunanagar	
Panchkula	Jind
Palwal	
Mewat	

**Table-3:** Agroclimatic zone-wise seroprevalence of BTV in Haryana as assessed by c-ELISA in cattle species.

Agroclimatic zone	Total number of sample assessed	Total number of positive samples	Positivity %
Eastern zone	303	232	76.56
Western zone	105	76	72.38

cELISA=Competitive enzyme-linked immunosorbent assay, BTV=Bluetongue virus

**Table-4:** Agroclimatic zone-wise BTV seroprevalence in buffaloes of Haryana as assessed by c-ELISA.

Agroclimatic zone	Total number of sample assessed	Total number of positive samples	Positivity %
Eastern zone	273	257	94.13
Western zone	122	110	90.16

c-ELISA=Competitive enzymelinked immunosorbent assay, BTV=Bluetongue virus

**Table-5:** District-wise seroprevalence of BTV in cattle and buffaloes of Haryana state.

Districts	Cattle	Buffalo
Ambala	35.00	95.00
Bhiwani	90.00	95.00
Faridabad	80.00	80.00
Fatehabad	70.00	80.00
Gurgaon	100.00	100.00
Hisar	53.30	91.66
Jhajjar	90.00	95.00
Jind	85.00	95.00
Kaithal	23.30	60.00
Karnal	89.47	100.00
Kurukshetra	66.60	50.00
Mahendragarh	100.00	100.00
Mewat	85.00	100.00
Palwal	100.00	100.00
Panchkula	90.00	100.00
Panipat	65.00	100.00
Rewari	70.00	93.33
Rohtak	100.00	100.00
Sirsa	45.00	80.00
Sonepat	95.00	95.00
Yamunanagar	58.33	100.00

Overall seroprevalence of BTV antibody in cattle and buffaloes for all 21 districts of Haryana state was found to be 75.49% and 92.91%, respectively. District-wise seroprevalence in cattle was observed as follows: Gurgaon (100%), Mahendragarh (100%), Palwal (100%), Rohtak (100%), Sonepat (95%), Bhiwani (90%), Karnal (89.47%), Jind (85%), Mewat (85%), Faridabad (80%), Panipat, Rewari, Kurukshetra, and Fatehabad (65-70%), Hisar and Yamunanagar (53-58%), Sirsa (45%), Ambala (35%), and Kaithal (23%), respectively.

In buffaloes of Haryana, the BTV seroprevalence was comparatively higher than in cattle, with the districts of Gurgaon, Karnal, Mahendragarh, Mewat, Palwal, Panchkula, Panipat, Rohtak, and Yamunanagar showing 100% seropositivity. Ambala, Bhiwani, Jhajjar, Jind, and Sonepat had seroprevalences of 95%. Rewari was 93% seropositive, while Faridabad, Fatehabad, and Sirsa each had seroprevalence of 80%. Kaithal and Kurukshetra were 60% and 50% seropositive, respectively.

## Discussion

BT, an arthropod-borne viral disease, mainly affects sheep and wild ruminants. Among all susceptible vertebrates, sheep are the main hosts [22]. Clinical signs mainly involve fever, nasal discharge, frothy salivation, excoriation of oral mucosa, and laminitis [23]. BTV viral genome is mainly surrounded by two major structural proteins such as VP3 and VP7 and three minor structural proteins such as VP1, VP4, and VP6. This in turn is surrounded by outer capsid containing two structural proteins VP2 and VP5. The VP7 and VP3 polypeptides are predominant and constitute more than 50% of the total BTV protein structure. The VP7 antigen has been found to be a highly conserved group-specific antigen [24].

Diagnosis of exposure to BTV mainly relies on detecting antibodies to a group-specific antigen. The AGID test is the most widely used assay for this purpose [25]. Although AGID is simple and rapid to perform, it is insensitive and gives cross-reactivity with other *orbiviruses* like EHDV. The use of monoclonal antibodies against BTV group-specific protein VP7 in c-ELISA can overcome this problem [26]. The serological response in BTV infection appears usually 7-14 days post-infection, and antibodies are generally long-lasting.

In India, the occurrence of BT between different parts of the country is dependent on the rainfall, with maximum number of outbreaks occurring during the North-East monsoon, followed by South-West monsoon [27]. In the study presented here, seroprevalence of BTV in cattle and buffalo was found to be 75.29% and 93.41%, respectively. Buffaloes exhibited higher seroprevalence than cattle. Serological evidence of BTV infection in local and exotic cattle breeds and buffaloes has been reported earlier from several states of India [28,29], indicating inapparent BTV infection in both cattle and buffaloes, although there are no reports of clinical BT in these animals in India.

A BT seroprevalence of 58.33% was observed in buffaloes in Gujarat [30]. In earlier studies by Raut *et al*. [31], a high seroprevalene was observed in cattle and buffalo of Maharashtra with 89.80% and 80%, respectively. However, in the southern state of India in Kerala, Arun *et al*. [32] reported that the percentage positivity in cattle was 6.9% which was below the overall prevalence of the disease previously reported in Kerala. Joarder *et al*. [33] screened a total of 313 animal serum samples (sheep - 68, goat - 195, and cattle - 50). 58.82% of sheep, 31.79% of goat, and 70.00% of cattle serum samples were found positive for the presence of antibodies for BTV. The prevalence of anti-BT antibodies in different agroclimatic zones ranged between 31 and 50%. This study revealed high seroprevalence of BT in cattle, sheep, and goats in Assam, north-eastern state of India. Bhagat *et al*. [34] tested a total of 510 goat serum samples from different part of Gujarat (western part of India). Among the samples tested, 292 (57.25%), 202 (39.61%), and 135 (26.47%) samples were found seropositive by c-ELISA, i-ELISA, and AGID test, respectively. Tigga *et al*. [35] studied serum samples of different small ruminants from Jharkhand (an eastern state of India) for the presence of BTV antibodies. Of the 480 serum samples tested, 83/190 (43.68%) sheep, 91/210 (43.33%) goats, and 46/80 (57.50%) cattle were found positive. The results of a study by Tigga *et al*. [35] showed slightly higher seroprevalence, although not significantly at two degrees of freedom (5%), in cattle than sheep and goats in different agroclimatic zones of Jharkhand. Sharma *et al*. [36] estimated the seroprevalence of antibodies to BTV among domestic ruminants of Grenada. They tested 928 sera samples (cattle - 133, goat – 314, and sheep - 481) using c-ELISA. They found that the overall BTV seroprevalence was 78.4% (95% confidence interval [CI] ± 2.65). The seropositivity of ovine, caprine, and bovine was found to be 71.7%, (95% CI, 67.67-75.73%), 80.2% (95% CI, 75.79-84.61%), and 98.5% (95% CI, 96.43-100.57%), respectively.

The high seroprevalence of BTV in cattle and buffalo population of Haryana state showed their prior exposure to BTV. The free movement of animals from the adjoining states of Rajasthan and intermixing with other animals for grazing (favoring two-way transmission of virus) along with an increase in the number of adult *Culicoides oxystoma* (the north Indian vector for BTV) during the rainy season overlaps with the spread of BTV in northern India. Moreover, proper surveillance program based on sero-monitoring can help forecast the possible future outbreaks. Attempts of isolation of the virus from the blood of the seropositive animals of Haryana and serotyping of the virus were not successful. There is no current BTV vaccination program in North India. Future, owing to the presence of large number of non-cross-protecting BTV serotypes circulating in the area, the control of disease in the region is difficult to achieve.

However, recent developments of inactivated or subunit vaccines may in the future help to control the disease in the state.

## Conclusion

Overall seroprevalence of BTV specific antibody in cattle and buffaloes for all 21 districts of Haryana state was found to be 75.49% and 92.91%, respectively. The prevalence of BTV in different agroclimatic zones ranged between 72-77% and 90-94% for cattle and buffalo, respectively. In buffaloes, the BTV seroprevalence was detected to be comparatively higher than in cattle. Conclusively, the study showed the BTV circulation is quite wide spread in cattle and buffalo populations in the Northern part of India.

## Authors’ Contributions

SM, AT, and DC: Analyzed the data. SM, AT, DC, and AD: Drafted the manuscript. SM, NK, NSM, VK, and NB: Contacted the officials of State AH department and collection of samples. SM, NK, AT, NSM, and AK: Provided reagents and materials. AT, KB, AD, and NB: Conducted the experiments. SM, DC, AD, AK, NK, and NSM: Proof read the manuscript and provided the guidance. All authors read and approved the final manuscript.
